# Research Progress of Plasmonic Nanostructure-Enhanced Photovoltaic Solar Cells

**DOI:** 10.3390/nano12050788

**Published:** 2022-02-25

**Authors:** Adnan Ali, Fedwa El-Mellouhi, Anirban Mitra, Brahim Aïssa

**Affiliations:** 1 Qatar Environment and Energy Research Institute (QEERI), Hamad Bin Khalifa University (HBKU), Qatar Foundation, Doha P.O. Box 34110, Qatar; adali@hbku.edu.qa (A.A.); felmellouhi@hbku.edu.qa (F.E.-M.); 2 Department of Physics, Indian Institute of Technology Roorkee, Roorkee 247667, India; anirban.mitra@ph.iitr.ac.in

**Keywords:** plasmonics, nanostructures, light trapping, lithographic techniques, silicon solar cell, organic solar cell, perovskite solar cell, simulation and modeling

## Abstract

Enhancement of the electromagnetic properties of metallic nanostructures constitute an extensive research field related to plasmonics. The latter term is derived from plasmons, which are quanta corresponding to longitudinal waves that are propagating in matter by the collective motion of electrons. Plasmonics are increasingly finding wide application in sensing, microscopy, optical communications, biophotonics, and light trapping enhancement for solar energy conversion. Although the plasmonics field has relatively a short history of development, it has led to substantial advancement in enhancing the absorption of the solar spectrum and charge carrier separation efficiency. Recently, huge developments have been made in understanding the basic parameters and mechanisms governing the application of plasmonics, including the effects of nanoparticles’ size, arrangement, and geometry and how all these factors impact the dielectric field in the surrounding medium of the plasmons. This review article emphasizes recent developments, fundamentals, and fabrication techniques for plasmonic nanostructures while investigating their thermal effects and detailing light-trapping enhancement mechanisms. The mismatch effect of the front and back light grating for optimum light trapping is also discussed. Different arrangements of plasmonic nanostructures in photovoltaics for efficiency enhancement, plasmonics’ limitations, and modeling performance are also deeply explored.


**Table of Contents**
Abstract:……………………………………………………………………………………………11. Introduction……………………………………………………………………………………12. Fundamentals: Physical Properties of Surface Plasmons…………………………………33. Plasmon Thermal Effects………………………………………………………………………54. Plasmonic Nanostructure Top-Down Fabrication Techniques……………………………64.1. Lithographic Fabrication Techniques………………………………………………………74.2. Nonlithographic Fabrication Techniques…………………………………………………75. Plasmonic Nanostructures for Light Trapping……………………………………………86. Mechanisms of Plasmonic Enhancement Effect……………………………………………97. Nanoparticle Material, Size, and Shape Effects……………………………………………107.1. Nanostructures at the Front Surface………………………………………………………117.2. Plasmonic Back Reflectors…………………………………………………………………127.3. Mismatch of the Front and Back Light Grating for Optimum Light Trapping………148. Different Arrangements of Plasmonics Nanostructures in PV and Mechanisms………169. Modeling the Performance of Plasmonic Solar Cells………………………………………259.1. Device Designs Using Propagation of EM Waves………………………………………269.2. Plasmonic Systems Based on Light Localization…………………………………………269.3. Problems Related to Light Scattering………………………………………………………2710. Summary………………………………………………………………………………………27

## 1. Introduction

The power of photovoltaics is continuously increasing, going from the current 800 GW worldwide to a predicted 1.3 TW by 2023 [1]. This rapid progress is mainly driven by improvement in solar cell materials and performance, and by the PV module power conversion efficiencies, reduced manufacturing, costs and the realization of levelized electricity costs, which are generally lower now than other energy sources. Silicon solar cells (Al-BSF) have held a market share of 70–90% over the last decades [2].

A key challenge is to increase the annual production of PV modules by 2040 to 3–4 TW annually [2]. Increasing efficiency is of key importance, as it reduces the required amount of energy-intensive materials such as c-Si and module glass, thereby reducing the energy payback time [3]. The silicon PV industry has many options to drive the efficiency of single-junction silicon solar cells to a practical technical limit of about 27.5% in laboratory and 26% in production [4]. The existing global photovoltaic solar cell market is 90% c-Si based solar cells, while the other 10% comprises perovskite solar cells (PSCs); dye-sensitized solar cells (DSSCs); CdTe, CIGS, µc-Si:H, and a-Si:H cells; etc. [5,6,7]. To fulfill global energy demand from photovoltaics, enhancements in light conversion efficiency and cost reduction are the main research targets. Four processes control solar energy harvesting, namely, light absorption, charge separation, charge migration, and charge recombination [8]. To enhance conversion efficiency and decrease cost, a nanophotonic approach for light entrapment has been explored. Different techniques have been applied to enhance light absorption in the active layer [9,10,11,12]. Usually, absorption of sunlight can be enhanced by increasing the thickness of the active layer. However, it is possible, because of the nanostructuring/nanopatterning in a solar cell active layer, to entrap light and increase the active layer optical thickness. Optical absorption enhancement provides the freedom to decrease the thickness of active the layer; this decrease has a direct effect on the cost. Furthermore, optical absorption is enhanced via an increase in diffusion length and high open circuit voltage. Nanophotonic structures also contribute to photostability and long-term yielding stability [13,14]. Similarly, organic photovoltaic cells (OPVs) have many advantages, such as low cost, light weight, and mechanical flexibility. OPVs suffer mainly from relatively low carrier mobility and small diffusion length [15]. Therefore, to improve the charge carrier diffusion and extraction, a very thin active layer (≤ 100 nm) is needed to minimize the recombination effect [16]. However, thinning the active layer reduces the light absorption and thus results in lower PCE. Therefore, to enhance light absorption and PCE of OPVs with a thicker active layer, the surface plasmon resonance effect might be utilized by introducing metallic nanoparticles [17,18,19,20,21]. Nanophotonic structures have the advantage of self-cleaning, because dust particles adversely affect solar cell efficiency by accumulating on the photovoltaic cell surface [22,23,24]. Nanostructures need to be engineered in such a way as to decrease optical losses at the front surface due to light reflection and enhance light transmittance.

For this purpose, in the industry, the main nanostructures used are inverted pyramid or upright structures [23,25,26,27] or random textures [28,29,30] with a distinctive typical period size of 3–10 µm applied primarily for c-Si solar cell texturing [31]. It was reported by Kumaravelu et al. [32] that for thin-film solar cells, where the thickness of active layer itself is a few microns/hundreds of nanometers, micron-scale structuring is not advantageous because it requires deep etching and easily creates defects in the active layer. Thus, the most suitable approach for light entrapment in thin film-solar cells is the application of nanostructures. Nanostructures for light entrapment are applied mainly as plasmonic metasurfaces and dielectric metasurfaces. Plasmonic metasurfaces are made on the basis of metallic meta-atoms, the optical responses of which are driven by the plasmon resonances supported by metallic particles. For dielectric metasurfaces, the unit structure is constructed with high-refractive index dielectric resonators such as silicon, germanium, or tellurium that can support electric and magnetic dipole responses based on Mie resonances. The responses of plasmonic and dielectric metasurfaces are relevant to the characteristics of unit structure, such as dimensions and materials. One can manipulate the electromagnetic field of light waves scattered by metasurfaces by designing the dimension parameters of each unit structure in the metasurfaces [33]. Chattopadhyay et al. [34] reported that by introducing nanostructures at the surface, light absorption in thin-film solar cells could be improved; this approach was more promising than micron-sized textures at the c-Si solar cell surface. The reason for this improvement is that nanostructure features are less damaging to the substrate, because deep etching is not needed [35]. Furthermore, in subwavelength nanostructures, reflections are curtailed beyond the Yablonovitch conventional limit, and enhanced optical path length can be obtained, as reported by Yu et al. [36]. There are numerous nanostructures with which light can be trapped in thin-film solar cells. The most used approaches for light trapping are plasmonic nanoparticle structures [37,38], random scattering surfaces [28], periodic nanograting [39,40], nanowires [41], and photonic crystal structures [28,42].

**Figure 1:**
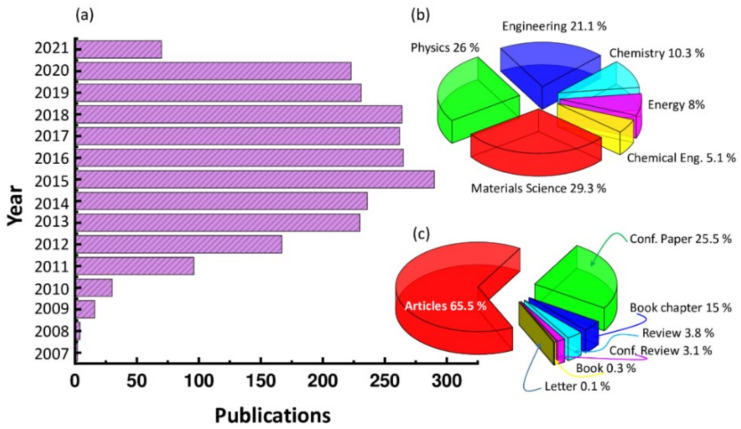
(**a**) Recent publications referred to herein related to the field of “plasmonics plus solar cell” together with distribution of these publications (**b**) per field and (**c**) per type of document. Data were collected from Scopus, an expertly curated abstract and citation database-based information service.

Plasmonic nanostructures applied to solar cells have emerged as a growing field of research in the last years, with a huge increase between 2007 and 2021 (with a peak recorded in 2015), as witnessed by the numbers of published works reported in Figure 1. These data were extracted from the Scopus database with the combined “plasmonic” and “solar cell” search terms.

In this review, we summarize up-to-date published work related to plasmonic nanoparticle applications in PV solar cells, both organic and inorganic, and how these applications affect device performance with different nanomaterials, sizes, shapes, combinations, and locations/placement in different layers of the device. It is also reported that the mechanisms through which the device performs change with these parameters.

## 2. Fundamentals: Physical Properties of Surface Plasmons

**Figure 2:**
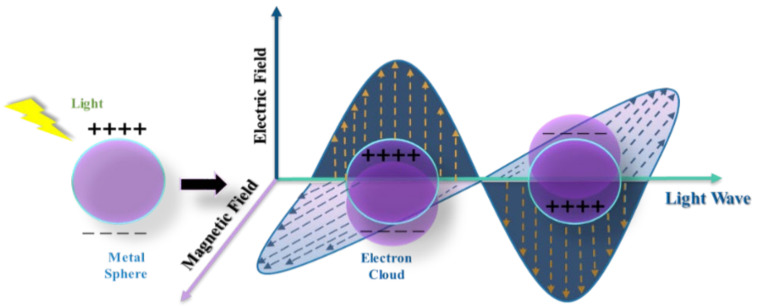
The electrons in a metal can careen like a jelly, pulled back by the attraction of the positive metal ions that they leave behind. Adapted from refs. [43,44].

Surface plasmon resonance (SPR) in solid state physics is known as a collective oscillation of electrons. The SPR phenomenon occurs when electromagnetic (EM) radiation is incident on a noble metal’s nanoparticle (NP) surface. This triggers a coherent oscillation of the electron cloud at the NP surface and leaves behind positive ions to vacillate like jelly. This happens because of the inherent nature of the alternating EM field of incident radiation. As a result of the coherent oscillation in the alternating field, the electron cloud distributes on the surface and away from nuclei. A restoring force is generated because of Coulombic attraction (see Figure 2). Usually, this phenomenon takes place at the interface of negative- and positive-permittivity materials.

Localized surface plasmons (LSPs) are the nonpropagating excitations of the conducting electrons coupled with EM field. The curved surface of the spherical nanoparticle applies a restoring force, which produces resonance in the electrons. This is called a localized surface plasmon resonance.

These modes occur because of the scattering issue of the small, subwavelength conductive NPs in the alternating EM field. As a matter of fact, this resonance for gold (Au) and/or silver (Ag) NPs occurs in the visible range of the EM spectrum. Therefore, one might see bright colors of particles in reflected and transmitted light spectroscopy because of the enhanced light absorption and scattering due to the resonance.

**Figure 3:**
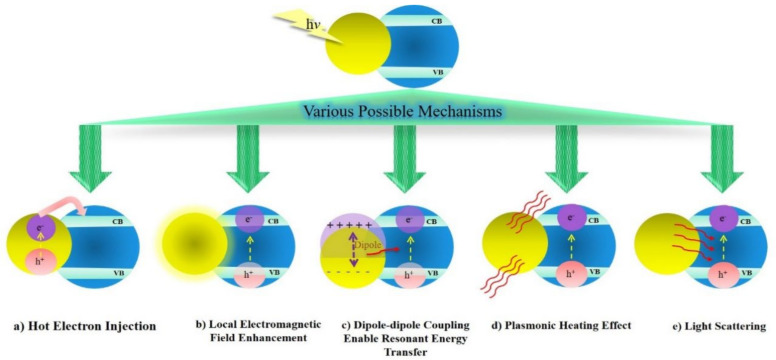
Mechanisms of LSPR photoactivity in semiconductors [54]. Adapted from ref. [54].

There are various localized surface plasmon resonance mechanisms of photoactivity by which metallic nanostructures may intensify semiconductors’ photocatalytic behavior [45,46,47]. Globally, five mechanisms explain exhaustively how metallic SPR enhances semiconductor photoactivity: (i) hot electron injection (or direct electron transfer [48,49]); (ii) local EM field enhancement (or light concentration [50]); (iii) dipole–dipole coupling enabling resonant energy transfer (or plasmon-induced resonant energy transfer [51]); (iv) the plasmonic heating effect [52]; and (v) light scattering [53]. These master processes are summarized schematically in Figure 3.

## 3. Plasmon Thermal Effects

**Figure 4:**
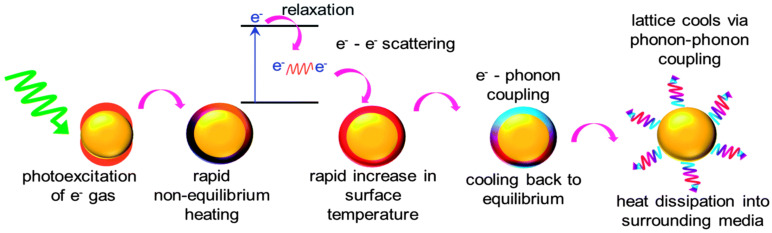
Description of photothermal light to heat conversion by plasmonic nanostructures [55]. Reprinted with permission from Ref. [55], Copyright 2014, The Royal Society of Chemistry.

The oscillation of the electron cloud over metallic NPs during SPR may greatly increase the surface temperature. This effect has been formulated as “plasmonic heating” and is depicted step by step in Figure 4 [55]. In sum, electrons undergo energy loss due to electrical inherent resistance. Defects in the crystal lattice structure, which are often present in nanoparticles, induce additional resistance. Vibrational energy is hence induced because of the energy lost by the electrons in the crystal lattice. This leads to a temperature increase. This increase in temperature can be very dramatic, e.g., several hundred degrees Celsius or up to the melting point of the material. This is most likely to happen when photons with a wavelength corresponding to the LSPR interact with the metal NPs [55,56,57].

Lalisse et al. [58] calculated the potential for plasmonic heating of various metal nanoparticles [58]. In the UV–visible region, plasmonic resonance for Au, Ag, and Cu occurred around 520, 360, and 300–550 nm, respectively. Of these metals, Au was expected to have the lowest ability to generate heat. However, these calculations were for pure metal nanoparticles. Both silver and copper oxidize rapidly in air, which considerably lowers their applicability. Gold, on the other hand, remains inert to oxidation even at nanoscale size, and hence, its plasmonic heating capacity has attracted additional interest. The other two studied materials were TiN and ZrN, and both exhibited true potential for plasmonic heating [59,60].

Because of the optical absorption associated with plasmonics resonance, heat is generated from metallic nanoparticles (i.e., through the conversion of EM field’s energy by resonant light illumination). This heat-generation process is activated by optical absorption and involves photon energy absorption as well as heat transfer from the nanostructures to the surrounding medium.

Wu et al. [61] showed that plasmonic nanostructures improved optical absorption. In addition, the group studied the thermal effects that took place and the associated heat transfer between nanostructures.

Plasmonic nanostructures’ dimensions and composition can explain the local and overall heat generation mechanisms. Furthermore the shape of plasmonic nanostructures plays a key role in distributing the temperature over the volume (or surface) and the overall temperature efficiency [62,63].

**Figure 5:**
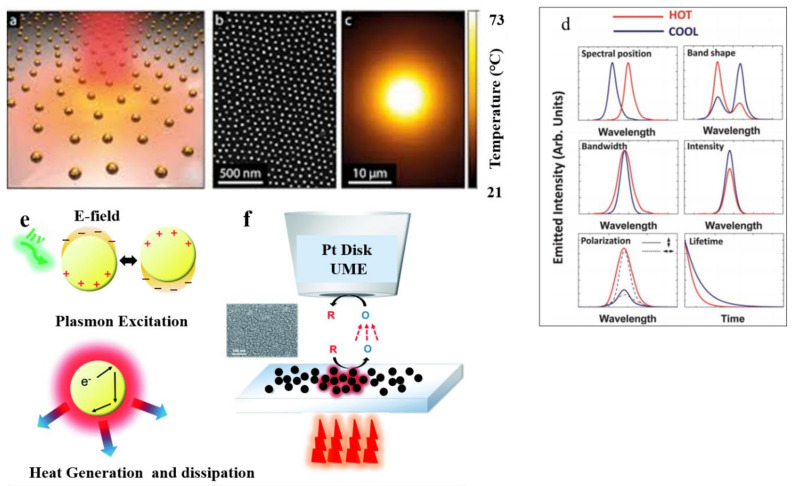
(**a**) Representation of glass decorated with gold nanoparticles. (**b**) SEM analysis of gold nanoparticles. (**c**) Gold nanoparticles after illumination temperature profile [77]. Reprinted with permission from [77], Copyright 2018, EDP Sciences. (**d**) Possible effects due to temperature increase are schematically represented. Effects due to temperature increase are depicted as red lines. (**e**) Presentation of (top) excited surface plasmons and (bottom) the resulting plasmonic nanoparticle surface temperature increase. (**f**) SECM setup with Au nanoparticles on a substrate, schematically depicted with an SEM image [75]. Reprinted with permission from [75], 2018, The Royal Society of Chemistry.

The groups of Willets [64], Mayer [65] and Zeng [66] reported that the LSP phenomenon may be strongly supported by metallic NPs showing higher numbers of free electrons (such as Au and Ag), as they can interact more efficiently with light and thereby produce collective oscillations at the surface. Furthermore, plasmon decay could occur radiatively through re-emitted photons [67] or nonradiatively via electron–electron and electron–phonon collisions. This conversion of energy produces heat [68,69]. This produced heat dissipates, and the local temperature rises in the surrounding area (see Figure 5a). Various parameters affect the heating efficiency, including light intensity, wavelength, light polarization, and especially plasmonic nanostructure geometry [65]. This increase in temperature is critical and needs to be measured accurately, as it must be accounted for when designing photothermal devices.

The techniques employed for measuring this temperature increase include scanning thermal microscopy [65], quadric-wave shearing interferometry [68], surface-enhanced Raman scattering [70], resistance measurements [71], thermographic phosphors [57], refractive index variations, infrared radiation, X-ray absorption spectroscopy, and microwave spectroscopy of nanodiamonds. In fact, it is rather easy to trigger a temperature increase temperature in Au NPs, for example, just by appropriately shining light on it. However, one challenge is how to control this increase quantitatively and accurately. Indeed, it is not straightforward to probe temperature at micro- and/or nanoscales. After the success of thermoplasmonics, probing temperature at the nanoscale was achieved by scanning thermal microscopy (SThM). In the SThM method, a nanometric thermocouple is put at the top of an STM tip to scan the sample. However, this technique is aggressive and not always appropriate. Therefore, fluorescence measurements techniques for plasmonics temperature microscopy have been introduced [72]. The idea behind these techniques is to use the correlation between fluorescence and temperature. In fact, most fluorescence properties are governed by temperature, including intensity, spectrum, lifetime of the excited states, and polarization anisotropy. Therefore, resonance is likely to scatter fluorescent molecules at the locality of metallic nanoparticles. Thus, the mapping of fluorescence reflects that of the temperature of the surface. Figure 5b,c display a typical example of temperature measurement performed on Au NPs by a using a label-free microscopy technique, which uses a quantitative wavefront sensing principle [73,74]. Scanning electrochemical microscopy (SECM) [75] is another photothermal measurement method, in which the increase in substrate temperature is measured after it takes place through light irradiating plasmonics. Using plasmonics metal NPs with an electroactive nature, particle–solution interfaces facilitate heterogeneous reactions and thereby allow electron transfer [76]. The photothermal effects can affect the process, increase the rate of mass transport of the redox molecules, and produce an equilibrium potential shift in the nanoparticle electrode. For measuring these effects, SECM can be used efficiently (see Figure 5e,f) [76].

## 4. Plasmonic Nanostructure Top-Down Fabrication Techniques

**Figure 6:**
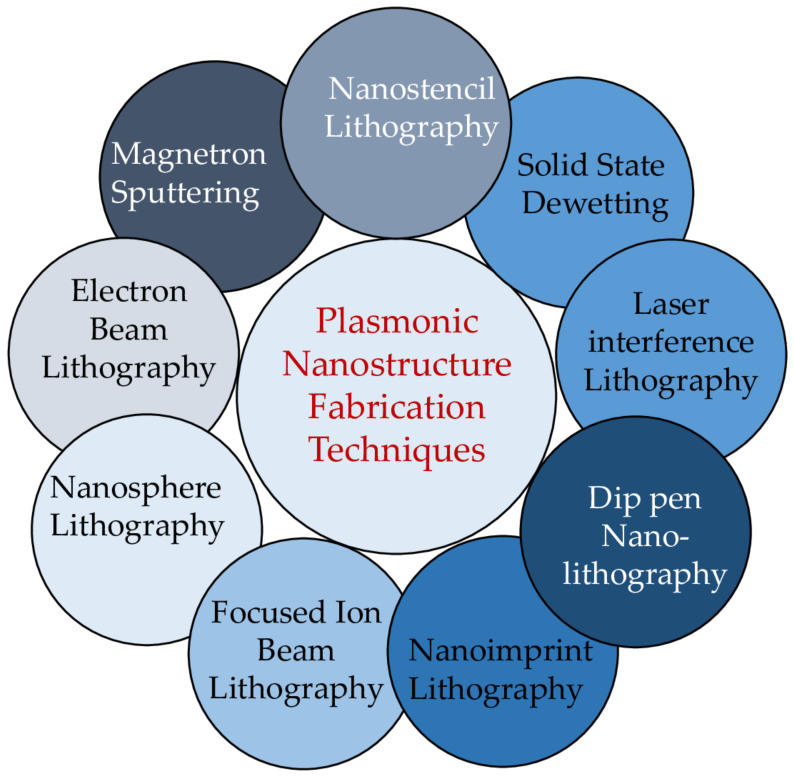
Plasmonics nanostructure top-down fabrication techniques.

To make use of plasmonic coupling effects for field enhancement, very accurate control over the dimensions of metallic nanoparticle arrays/structures is required. For local field enhancement, the common approach is to use metallic nanoparticle colloidal aggregates [78], and to benefit from the resonant LSP mode, tailoring the EM field and the reproducibility of the different plasmonic nanostructures to get the same output is required, since randomly oriented aggregates/nanoparticles have demonstrated limited efficiency. To this end, different fabrication techniques have been explored and are summarized in Figure 6. Currently, due to the huge advancement in nanofabrication technologies, the control over plasmonic nanostructures at the nanometer scale is very precise and accurate.

### 4.1. Lithographic Fabrication Techniques

The main tool for transferring patterns from substrate to substrate is lithography. Commonly used lithographic fabrication techniques for fabricating plasmonic nanostructures are electron beam evaporation (EBL) [79,80], focused ion beam (FIB), nanosphere lithography (NSL), laser interface lithography, stencil lithography, stamping or nanoimprint lithography (NIL), and dip-pen nanolithography (DPN), as shown in Figure 6. To achieve patterns with features smaller than 100 nm and subwavelength periodicities, EBL would be the appropriate choice. EBL can realize patterns as small as ~10 nm with high precision and reproducibility. EBL and lift-off processes are used together. The EBL process has been used successfully to fabricate plasmonic nanostructures including nanopyramids, nanogratings, nanocylinders, and nanorings [79,80,81,82].

Another convenient technique is FIB lithography. It is used mainly in optically thick metal films to fabricate nanohole array and slot structures. Khan et al. [83] studied nanohole arrays fabricated in metal film and involving Fano resonances (i.e., resonant scattering that results in an asymmetric line shape) propagating SPPs and LSPPs successfully. They achieved nanoscale features with 4:1 aspect ratio accuracy in a 30–60 nm-thick metal of Au with the FIB milling technique using He/FIB milling.

NSL is another vigorous and inexpensive fabrication technique. It is employed to fabricate ordered 3D nanostructures on a large area, offering a high degree of freedom over the tuning of the nanostructures’ geometry, periodicity, and material. Using this lithographic technique, nanometer-scale 3D nanostructures with controlled periods, height/hole depths, and inner/outer diameters and pitches. NSL has been successfully used for nanotowers (solid/hollow) and nanorings/nanodiscs fabricated on different substrates [84,85,86].

Laser interference lithography (LIL) is another fabrication tool technique and is maskless. The pattern is recorded on a photoresist material by the incident laser beam’s interference. Unlike EBL, LIL can efficiently fabricate 2D patterns on a large surface area. Different structures can also be achieved by playing with the laser operating parameters [87,88].

Stencil lithography [89,90] is a resistless technique that employs a shadow mask to achieve micro- and nanometer-scale surface structuring. A stencil is clamped on the substrate, and material is deposited in an evaporating chamber through the stencil’s apertures. This technique can also be used for implantation and etching. Two main types of stencil lithography are used based on the material’s properties: (i) rigid mask stencil lithography, which uses Si and SiN_x_ masks, and (ii) flexible mask stencil lithography, which employs flexible masks such as polyimide films, PDMS membranes, and photoresist layers [91,92]. Stencil lithography is also categorized based on the motion involved. Dynamic stencil lithography involves motion of the stencil relative to the substrate during the deposition or in between the deposition steps and allows micro-/nanopatterns, multilayers, and different materials for in situ fabrication. Static stencil, as indicated by its name, involves a static substrate and stencil [89,90,93].

Stamping, or nanoimprint lithography (NIL) [94], is another technique for large-area nanostructuring that offers high throughput with high precision and reproducibility. Unlike lithographic techniques using light diffraction or beam scattering, in NIL, a resist is directly deformed mechanically, and this faculty allows fabricating high-resolution features (even at the subwavelength scale). NIL is a very promising technique for large-scale plasmonic structure integration and can be easily extended to complex structures [95,96].

Among the direct-write developed techniques, dip-pen nanolithography (DPN) [97] is a very powerful nanolithographic process like NIL. Instead of stamping, DPN uses a nanoprobe to pattern different surfaces with a resolution down to 15 nm. DPN can be used either in soft materials (polymers, small organic molecules, proteins, DNA) or hard materials (metal oxide NPs, semiconductors, sol–gels) [98,99,100,101].

### 4.2. Nonlithographic Fabrication Techniques

For plasmonic nanostructures, the most used nonlithographic fabrication techniques are solid state dewetting (SSD) and magnetron sputtering, as shown in Figure 6. Solid state dewetting [102] (SSD) is a technique in which a thin film on a substrate disintegrates and forms into separated objects, such as droplets, stripes, and pillars. Depending on process parameters (temperature, time, etc.), random and ordered patterns can be obtained by using either a topographically controlled substrate or a substrate with a controlled surface tension [103,104]. Ordered particles arrays and complex patterns can also be achieved by SSD using prepatterning of the films [105,106].

The template-assisted magnetron sputtering technique can be used to fabricate plasmonic nanostructures. Qin et al. [107] used magnetron sputtering to deposit a metallic thin film on a nanocup template with well-separated and well-defined features. By varying DC magnetron sputtering deposition time, different shell thickness were obtained. This technique was used to tune the plasmonic resonance. Similarly, it can be used for different nanostructured templates such nanoholes [108] and nanostructures arrays [109] as well as for SERS application [110].

## 5. Plasmonic Nanostructures for Light Trapping

Solanki [111] reported that the energy from the sun reaches the earth surface contains 48% visible, 43% infrared, and 7.5% ultraviolet radiation. Therefore, to harvest solar energy, both regions of the solar spectrum should be targeted. To absorb solar radiation at large with a photovoltaic device, the front and back layers should be designed accordingly. The front surface should be designed to enhance the light transmission into the device, while the surface of the back layer (i.e., facing inward) should be designed to reduce this transmission.

The performance of the thin-film solar cell is typically limited by the low light absorption coefficient and thickness of the absorption layer. Therefore, enhancing the light absorption and its conversion efficiency into electrical current may significantly reduce the cost of production of PV energy. Key sets of methodologies have been developed in order to achieve this goal, including the use of nanostructures, such as gradual refractive index matching, localized plasmon resonances, surface plasmon polariton modes, and coupling incident light into guided modes [112].

Using plasmonic nanostructures for photocurrent enhancement in solar cells has some technical barriers that are important to consider, such as (i) parasitic absorption, i.e., photocarrier loss, which produces heat via nonradiative channels as reported by Santbergen et al. [113], Gee et at. [114], and Palanchoke et al. [30]. This can be controlled to some extent by manipulating the metallic nanostructures’ size, shape, and patterning. (ii) Xue et al. [115] and Du et al. [116] reported that plasmonic nanostructures incorporated in the absorption layer can act as recombination centers for carriers. This can reduce short circuit current and open circuit voltage [117]. This can be overcome by introducing a thin dielectric casing around the plasmonic nanostructures. (iii) Materials such as Au and Ag plasmonics metallic nanoparticles can be costly. Plasmonic nanostructures can broaden the absorption spectral regime in the photovoltaic material. On the other hand, where absorption cannot be enhanced, plasmonic nanostructures can affect the potential of the system.

**Figure 7:**
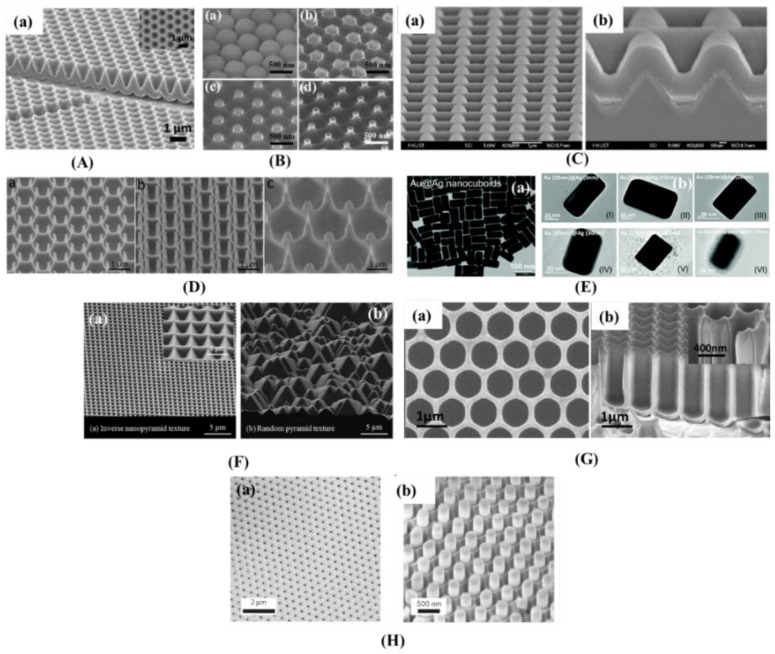
(**A**). (**a**) SEM of Au nanocone-coated template with 1 µm pitch and 1 µm depth (reprinted with permission from [24], 2012, John Wiley and Sons). (**B**) SEM of a 500 nm polystyrene nanosphere monolayer and Si nanorod array. (**C**) SEM micrograph (75° angle) of an ultrathin a-Si/c-Si tandem solar cell on the top surface and at the cross-section (reprinted with permission from [24], 2012, Elsevier). (**D**) SEM view (60° angle) of nanopillar structures with different pitches and heights (**a**,**b**). Cross-sectional view SEM of integrated nanopillar/nanowell structures. (Reprinted with permission from [124], 2009, Springer Nature). (**E**). (**a**) TEM images of Au@Ag NCs and individual Au@Ag NCs with different Ag shell thicknesses along with an individual Au@Ag@SiO2 NC (reprinted with permission from [127], 2010, American Chemical Society). (**F**). SEM of surface textures showing 2D grating of an inverse nanopyramid pattern and the industry standard random pyramid texture (reprinted with permission from [128], 2012, American Chemical Society). (**G**). (**a**) Top view and cross-sectional view SEM of the nanowell sample. (**H**). (**a**) SEM of an as-made anodic alumina membrane (AAM) with perfectly ordered pores along with a CdS nanopillar array after partial etching of the AAM (reprinted with permission from [124], 2009, Springer Nature).

**Figure 8:**
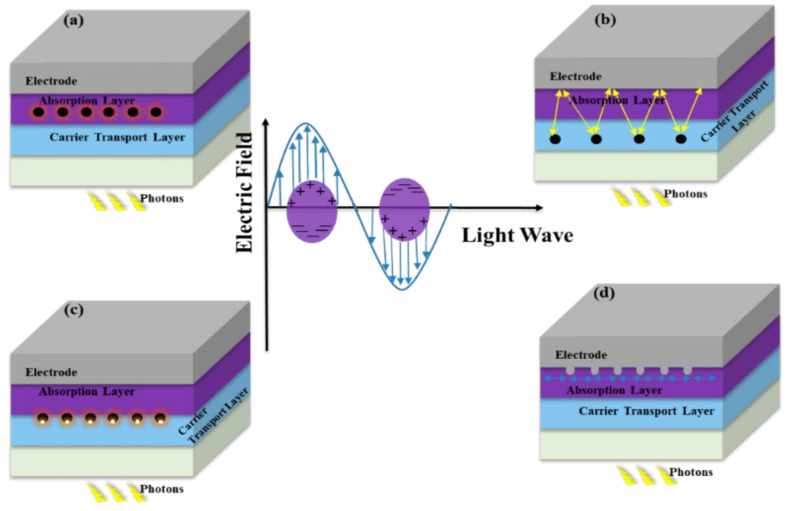
The graph in the middle explains the LSP mechanism in metallic NPs. Structural design of solar cells with plasmonic metallic nanoparticles. (**a**) LSPR enhancement by embedding nanoparticles in the absorption layer; (**b**) embedding nanoparticles for trapping light via the forward scattering effect in the charge carrier transport layer (CTL); (**c**) nanoparticles in the CTL that induced the enhancement of the electromagnetic field in the photoactive layer via the LSPR effect; (**d**) light trapping by the excitation of surface plasmon polaritons (SPPs) at the metal–semiconductor interface (nanostructured metal films placed on the back surface of a solar cell).

Numerous nanophotonic structures, including nanocones [24,118,119,120] (Figure 7A), Si-nanorods [121,122,123] (Figure 7B(a–d)), nanospheres [124,125,126] (Figure 7C(a,b)), nanopillars and nanowells [24,124,125,126] (Figure 7D(a–c)), nanocuboids (Figure 7E(a,b)), nanopyramids [9,26,27,127,128,129,130,131] (Figure 7F(a,b)), nanowells [125,127,128] (Figure 7G(a,b)), and nanopillar (Figure 7H(a,b)) have been extensively studied for improving the efficiency of solar cells. Dielectric, metallic, and absorber layers [132,133,134,135] can be applied as photonic nanostructures. Nanostructures can be incorporated in the solar cell in three different scenarios, as summarized in Figure 8: (1) at the front surface, (2) at the back surface, or (3) embedded into the active absorber layer.

## 6. Mechanisms of Plasmonic Enhancement Effect

In solar cells, light trapping can be enhanced by incorporating metallic nanoparticles, in which electrons can be excited at the interface of the metal and dielectric layers. An EM field then amplifies in the active layer under the SPR effect (see Figure 8). Mie theory explains this EM field amplification [136,137].

Various SPR mechanisms can be used to enhance plasmonics effects such as the near-field localized effect, the far-field scattering effect, waveguide mode, and plasmon-cavity mode [138,139,140,141,142]. By the far-field scattering effect, optical absorption enhancement can be obtained. This occurs because the incident photons’ optical path increases, and the reflection decreases. On the other hand, because of near-field SPR effect, absorption is enhanced. It is known that metallic nanostructures can efficiently limit the EM at the metal–dielectric interface and thereby intensify the SPR effect [143].

Many groups have demonstrated [144,145] that in metal nanoparticles, surface plasmons excite LSP, while at the interface of metals and semiconductors, they propagate SPPs (see Figure 8). Metallic nanoparticles can also be implanted into the active layer as subwavelength optical antennas. Through this configuration, the plasmonic near field can be coupled to enhance the light absorption in the surrounding media (Figure 8a) [146,147].

## 7. Nanoparticle Material, Size, and Shape Effects

**Figure 9:**
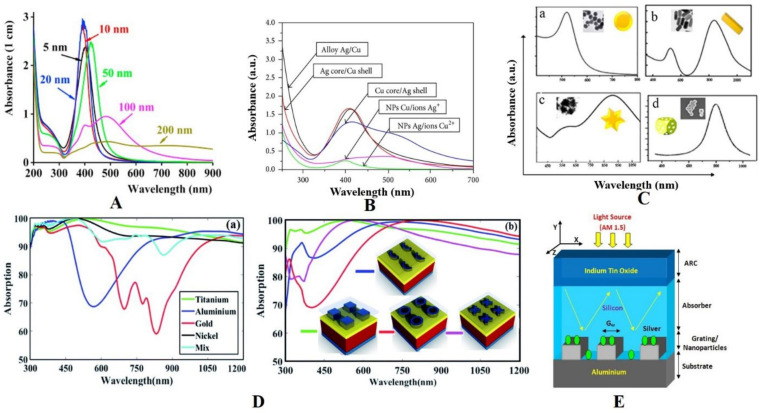
(**A**). Absorbance spectra of different sizes of Ag NPs [157]. (**B**). Absorbance spectra of bimetallic (Ag/Cu) nanoparticles [158] (reprinted with permission of Creative Commons Attribution License). (**C**). Various types of gold nanoparticles and their UV absorption (Reprinted with permission of Elsevier and Copyright Clearance Center): (**a**) spherical gold nanoparticles, (**b**) gold nanorods, (**c**) gold nanostars, (**d**) gold nanocages. [159] (**D**). Influence on optical absorption with (**a**) different materials and (**b**) different shapes of NPs [160] (Reprinted with permission from [160], 2019, Creative Commons Attribution—Noncommercial 3.0 Unported License.). (**E**) A schematic diagram of an ultrathin silicon solar cell representing 20 nm-diameter Ag NPs periodically substituted on the Al gratings to enhance the light-trapping mechanism [154].

For the case of plasmonics-based devices, key factors to investigate are related to the scattering and coupling effects [138,148,149,150] and revolve mainly around surface nanoparticles’ material, geometry, size, and distance from the active layer, as well as the medium’s refractive index, as depicted in Figure 9A–C. Pillai et al. [150] investigated how the normalized scattering cross-section (SCS) and sphere-shaped particles interacted in two different media, air and silicon. In the latter, because of plasmonics resonance, a clear redshift occurred (comparatively to air), in addition to a clear increase in the light trapping observed in the red and near-IR regions generated through the resonance peaks’ redshift and broadening [150].

The particle shape has a strong effect on the scattering efficiency, and it has been reported that with particle size of ~100 nm, a relatively high scattering efficiency can be achieved. Catchpole et al. [148] reported that cylindrical and hemispherical particles could contribute more to enhancing light absorption than spherical particles. In hemisphere geometries, the average spacing to the substrate is smaller than in sphere geometries, leading to effectual coupling of the scattered light and semiconductor substrates.

The plasmonics resonance peak is consistently related to a high light enhancement effect. However, it may be altered by the refractive index value of the adjacent material. For instance, for Ag and Au nanoparticles, plasmonics resonance appears at 350 and 480 nm, respectively. It redshifts to the 500–1500 nm range when these NPs are deposited onto SiO_2_, Si_3_N_4_, or Si [151,152,153]. As the distance between the nanoparticles and the absorbing layer increases, the light scattered into the absorbing layer decreases [148]. A dielectric layer can be used to prevent carrier recombination, which occurs at the metal surface. Indeed, compared with Au, Ag nanoparticles improve the light absorption [154], as shown in Figure 9D,E (and Ag is even cheaper than Au). On the other hand, Al nanoparticles have increased photon absorption by 28.7% when deposited at the front side of Si wafers, which is much a much higher increase than those reported for Au and Ag [155]. When coupling Al nanoparticles with SiNx antireflection coating, a huge enhancement of 42.5% in the photon absorption was achieved [156], paving thus the way for a cost-effective and highly efficient combination. Uhrenfeldt et al. [156] demonstrated that by depositing Al nanoparticle periodic arrays at the front of a thin Si film, an enhancement in the photogenerated current was obtained with respect to the reference cell.

### 7.1. Nanostructures at the Front Surface

**Figure 10:**
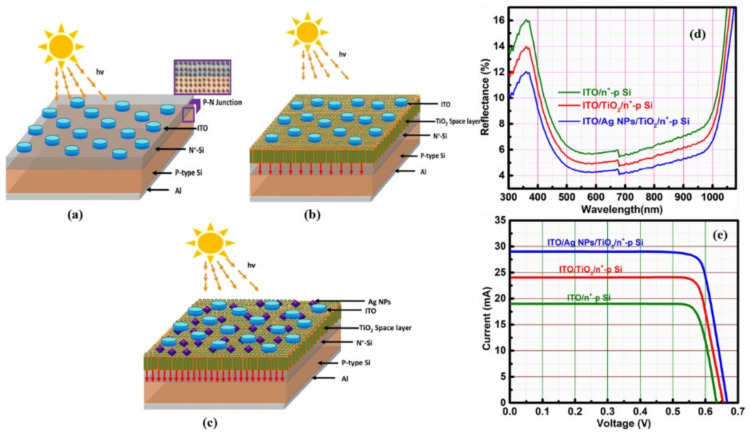
(**a**) Bare thin Si-based solar cell. (**b**) Si solar cell with TiO_2_ thin film as ARC. (**c**) Si solar cell with TiO_2_ film as ARC and Ag nanoparticles (NPs). (**d**) Reflectance spectra of the three prepared solar cell configurations. (**e**) I/V characteristics of the prepared solar cells [162]. (Reprinted with permission from [162], 2020, Springer Nature and Copyright Clearance Center).

The top layer of the solar cell is a critical location at which to reduce light reflection and losses and increase light trapping so that light with low and high wavelengths can enter into the cells. This can be achieved by the deposition of an antireflection coating at the top surface. To couple incident light into an absorption layer, metallic nanoparticles can be utilized as subwavelength scattering elements [161]. Localized surface plasmons can be generated by appropriately engineering metallic nanoparticles to efficiently scatter the light. Because of the scattering of the metallic nanoparticles deposited at the top surface, the optical path length of the light can be enhanced inside the absorption layer. Jain et al. [162] investigated TiO_2_ film of 30 nm thickness as an antireflection coating (ARC) along with plasmonic Ag NPs (90 nm). Plasmonic Ag NPs were applied at the front surface (Figure 10a–c). The incident light reflection was reduced by the TiO_2_ film on the top Si surface and Ag NPs thanks to the plasmonic effect, which improved light trapping (Figure 10d). This combination led to an enhancement in the power conversion efficiency (PCE) (Figure 10e). The PCEs of a bare Si solar cell, a Si-cell with TiO_2_ as ARC, and a Si-cell with TiO_2_ as ARC along with Ag NPs were measured as 9.53%, 12.58%, and 16.04%, respectively. Thus, the ARC/plasmonic combination enhanced the PCE by ~40%.

### 7.2. Plasmonic Back Reflectors

After letting more light enter the solar cell, the second important purpose is to enhance the optical path length of the photons in the active layer. To obtain this goal, the inner side of the back surface of the cell needs to be textured properly to make longer wavelengths scatter and diffract back into the active absorption layer, as shown in Figure 8d. A plasmonic nanostructure can be applied in the thin-film solar cell to harvest the maximum amount of trapped light [163,164,165]. This plasmonic nanostructure can be designed to absorb different wavelengths of light, such as those in the infrared [166,167,168] and visible range [169].

**Figure 11:**
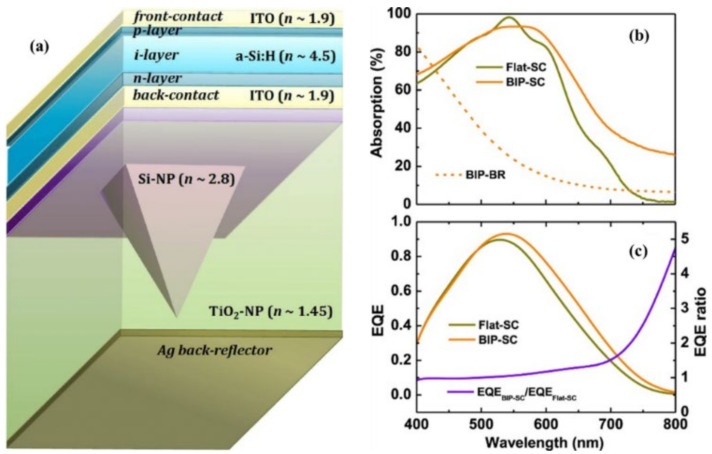
(**a**) Schematic illustration of a BLiS back reflector for n–i–p Si thin-film solar cells. The TiO_2_ -NP layer over the planar silver back reflector had inverted pyramid-shaped microcavities, which were further covered by a flat-topped Si-NP layer. (**b**) Total optical absorption by Flat-SC and BIP-SC devices (solid lines) and parasitic absorption by the BIP-BR (dashed line). (**c**) Measured EQE (left-hand side *y*-axis) spectra of Flat-SC and BIP-SC devices and EQE ratio (right-hand side *y*-axis) [173]. Reprinted with permission from [173], 2020, Creative Commons Attribution License (https://creativecommons.org/licenses/by/4.0, accessed on 4 February 2020).

Hungerford et al. [170] reported a back reflector based on Ag nanoparticles of hemispherical shape. With this, 72% absorption in the 400–600 nm spectral range was achieved. Crudgington et al. [171] reported on using a Ag nanoparticle array as a plasmonic back reflector in a thin-film solar cell. In the UV region at 140 nm spectral width, 80% absorption was achieved by the Ag array. Sun et al. [172] studied a new design for a thin-film solar cell back surface involving decorating the bottom surface of the silicon layer with Ag nanoparticles. By doing so, in the solar spectrum from 800–1200 nm, 50% of transmission losses were minimized. C. Sun et al. [11] suggested another design in which the back surface was textured with blazed grating to overcome the issue of transmission losses in the visible range in thin-film solar cells. This texturing solved the issue of transmission losses yet raised a recombination issue, which eventually decreased the overall efficiency. Desta et al. [173] reported on back reflector in a thin-film Si solar cell involving flat Ag with an overlying bilayer, which was a dielectric TiO_2_ layer with inverted nanopyramidal cavities, used to scatter light (Figure 11). With this approach, efficient light scattering was achieved. An increase in J_sc_ and an efficiency increase from 14% to 17.5% was reported.

**Figure 12:**
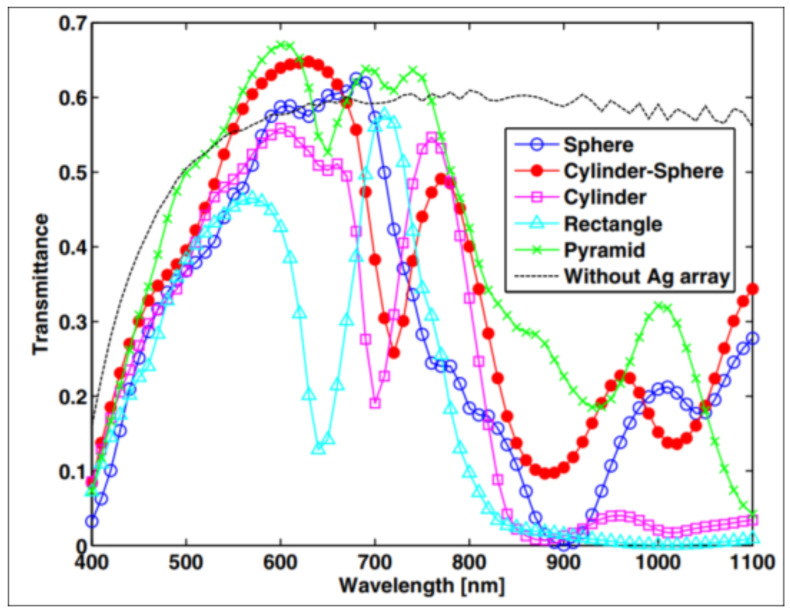
Bottom configuration: the silver nanoparticle arrays with individual particles shaped as rectangles or cylinders gave rise to the lowest transmittance curves [172]. Reprinted with permission from [172], 2012, American Chemical Society.

Sun et al. [172] demonstrated that light could be more efficiently prevented by cylindrical or rectangular Ag nanoparticles to transmit away from silicon than by other designs, as shown in Figure 12. This characteristic was ascribed to the interface area of Ag/Si, which was comparatively greater for cylindrical and rectangular nanoparticles. Therefore, they efficiently scattered light back into the silicon. This scattering effect was due to the excitation of the surface plasmons of Ag nanoparticles.

**Figure 13:**
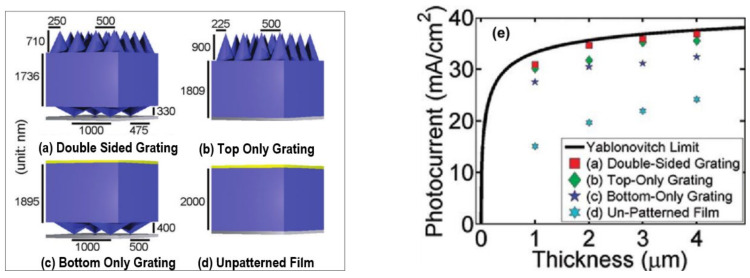
Three-dimensional silicon thin-film structures in air. From (**a**) to (**d**), blue represents silicon, gray represents a perfect electric conductor (PEC), and yellow represents nonabsorbing silicon nitride. The nanocones were made of silicon, as were the uniform layers, and they were placed in a two-dimensional square lattice either on the front or on the back surface of the film. (**a**) The optimized double-sided nanostructure. (**b**) The optimized top-only nanostructure. (**c**) The optimized bottom-only nanostructure with a thin layer of nonabsorbing silicon nitride on top. (**d**) The flat film with a thin layer of nonabsorbing silicon nitride on top [178]. (**e**) Photocurrents generated by structures as a function of their equivalent thicknesses. (Reprinted with permission from [178], 2012, American Chemical Society).

It was reported by Amalathas et al. and Eisele et al. [25,174] that periodic arrays of nanostructures as back reflectors of light constitute a highly promising methodology. They can help to couple the incident light and propagate in the absorber layer to harvest it at the fullest. The key to control the scattered light polarization and distribution lies in controlling the shape of the nanostructured periodic array. Optical path length within the absorption layer can be increased substantially by applying nanostructured periodic arrays as back reflectors. Haase et al. [175], Aissa et al. [176], Sai et al. [177], and Wang et al. [178] reported a wide range of range of nanostructure shapes, dimensions, and periodicities applied as back reflectors for light trapping. They explored how to optimize these parameters to trap and harvest the incident light efficiently in thin-film solar cells. In Figure 13a–d, four different cases are presented and then compared. Benefits of the double side microcone grating applied on an ultrathin c-silicon solar cell were studied by systematic rigorous coupled wave analysis (RCWA) [178].

The optimized double-sided grating structure yielded a photocurrent of 34.6 mA/cm^2^ at an equivalent thickness of 2 μm, which approached the Yablonovitch limit [179], as shown in Figure 13e. This methodology is appropriate to numerous thicknesses and is vigorous against metallic loss in the back reflector.

### 7.3. Mismatch of the Front and Back Light Grating for Optimum Light Trapping

Various other photonic crystal morphologies including nanopyramids, nanodomes, and nanowires have also been reported. Photonic crystal shape has strong effects when the size of the periodic array of the nanostructure is equal to or smaller than the wavelength of the incident light. Putnam et al. [180] demonstrated that the light trapping phenomenon has a limit dictated by the local photonic density of optical states. Therefore, trapping light can be substantially enhanced with photonic crystals for the absorption layer [181,182,183]. The numerous types of photonic crystals applied to solar cells include periodically arranged strips [184], nanodomes [185], nanopillars, nanoholes and nanowells [186].

**Figure 14:**
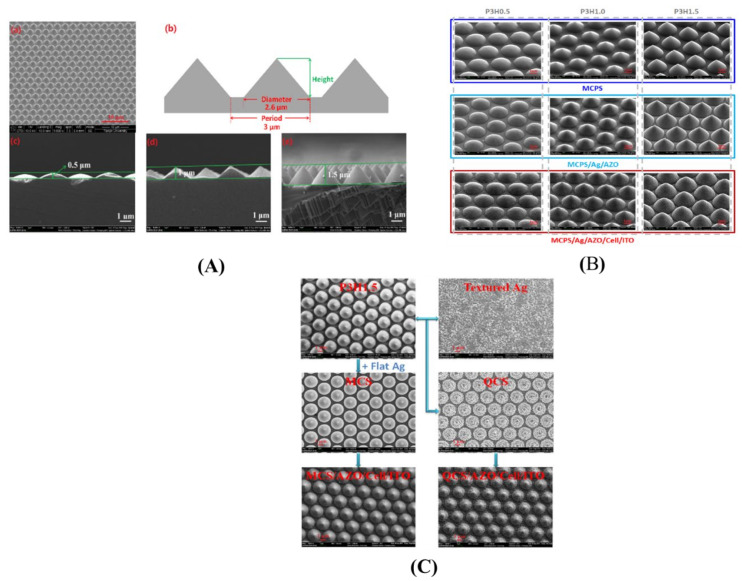
(**A**) (**a**) SEM image of a microcone-patterned substrate (MCPS) with a uniform microcone pattern; (**b**) schematic structure of the microcone pattern; (**c**–**e**) cross-sectional SEM of MCPSs with different H/P. (**B**) SEM images of MCPS (P3H0.5, P3H1.0, P3H1.5), MCPS coated with Ag/AZO, and a corresponding solar cell. (**C**) SEM images of microcone structure (MCS), quasicrystal structure (QCS), and MCS-based and QCS-based a-SiGe:H solar cells [28] (reprinted with permission from [28], 2017, Creative Commons CC-BY license).

Ding et al. [28] investigated a nanocone grating with architecture with a high aspect ratio and a dense periodicity of about 500 nm. This configuration was applied at the front surface as an antireflection coating, and it was successfully demonstrated that a substantial increase in absorption could be obtained. Once applied at the back surface, low-aspect ratio nanocones in lower-density nanocone grating also considerably improved the absorption inside the device (Figure 14). By implementing this grating at the front and the rear sides simultaneously, a guided coupling was achieved. For a 2 µm thin Si cell, the optimal periodicity for light tapping of the nanocones on the back surface was found to be 1000 nm.

Si-based nanowires arrays have also been applied for solar cell applications. Solar cells with radial p–n junctions containing Si nanowire arrays were demonstrated to offer wide band optical absorption properties and a higher collection of charge carriers. Nanowire array patterning, the aspect ratio, and the distance between the wires strongly govern both the light absorption and the charge carrier collection efficiency [180,187].

**Figure 15:**
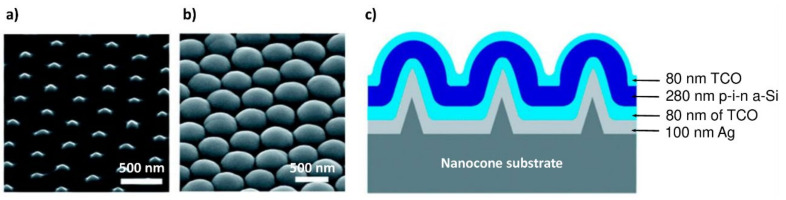
SEM images at 45° on (**a**) nanocone-patterned quartz substrate and (**b**) a-Si:H nanodome solar cells after deposition of all layers on nanocones (scale bar 500 nm). (**c**) Schematic illustration of the cross-sectional view of a-Si:H nanodome solar cells (Reprinted with permission from [185] 2010, American Chemical Society).

Zhu et al. [185] reported (Figure 15) that a-Si:H solar cells deposited on a substrate patterned with 280 nm-thick nanodomes enhanced the refractive index matching with air and increased absorption and optical path length. This combination of a-Si:H with nanodome configuration was very effective because a-Si:H absorbed most of the 400–500 nm wavelengths. Compared with the 65% absorption of the planar configuration, absorption of light increased to 94% in the 400–800 nm wavelength range with nanodomes, which in turn improved the PCE.

## 8. Different Arrangements of Plasmonic Nanostructures in PV and Mechanisms

In device structure, the incorporation of metal nanoparticles as plasmons can enhance device performance by different mechanisms depending on their placement. There are several ways to incorporate metallic nanoparticles, such as into the absorption layer, distributed in the charge transportation layer, applied onto the electrodes, or in between the layers as a sandwich structure. Table 1 summarizes some reported studies in which plasmonic nanoparticles were applied in organic solar cells [188] along with the recorded performance. Mainly nanoparticles of Ag, Au, Ag/Au (bimetallic) were incorporated in the organic PVs to harvest more light energy. Au in the active layer as a plasmonic nanoparticle performed better than Ag. Ag/Au bimetallic nanoparticles in the HTL also boosted the PCE. There is no straightforward mechanism in terms of specific nanomaterials as plasmonic nanoparticles, because there are different organic solar cell configurations. However, incorporation of nanoparticles in the active layer of the OPV is advantageous.

In Table 2, research involving incorporation of nanomaterials as plasmonic nanoparticles in metal-halide perovskite solar cells is summarized to understand the effect of said incorporation. There are many different configurations of PSCs, and each one has its own performance mechanism and explanation. In most cases, the plasmonic nanoparticles improved the photocurrent. To have high carrier generation rate, the amount of absorption in the active layer must be increased. The increase in PCE can be attributed to an increase in short-circuit current density (J_sc_) due to an increase in optical path length and absorption. NPs exhibit the ability to increase the optical path length of photons in the device via near-field and far-field enhancement (plasmonic scattering) and consequently may act to improve the photon-to-electron conversion efficiency (ΔIPCE) and PCE of PSCs. Reducing the thickness of the active layer reduces the amount of absorption, but this can be compensated by stimulating the plasmons of metal NPs. Since the thickness of the active layer and recombination are directly related, reducing the thickness of the active layer can have a positive effect on the recombination rate.

In Table 3, the plasmonics effects of nanoparticles in silicon solar cells are tabulated and the respective effects on cell performance and mechanisms are summarized. In thin-film silicon solar cells, plasmonic effects constitute an emerging technology with promising application in the solar cell fabrication industry. It uses the nanoscale properties of Ag, Au, Al, Ti, Cu, SiO_2_, etc. nanoparticles incorporated in the interface between the metal and dielectric contacts to enhance the light-trapping properties of thin-film silicon solar cells by increasing the absorption capacity and the generation of hot electrons, which in turn enhance the photocurrents in the solar cell. Plasmonic nanoparticles applied on the front (i.e., top) surface of the silicon solar cell were most effective and easily deposited.

## 9. Modeling the Performance of Plasmonic Solar Cells

**Figure 16:**
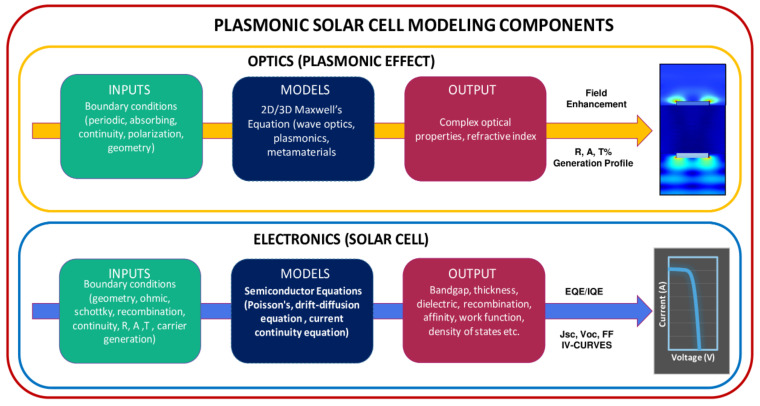
Plasmonic solar cell modeling components with respective input parameters, models, and output parameters required for device simulation.

Modeling of plasmonic solar cells (PSC) is vital for assessing the geometrical and operating conditions for optimal optoelectronic performance. In principle, modeling of PSCs is a multiphysics problem comprising optics and electronics [246], as shown in Figure 16. Optical physics is associated with the plasmonic structure and light propagation, while electronics is associated with the physics of solar cells, which is required for investigating carrier transport and extraction.

Electric field intensity, resonance modes, absorption (A), reflection (R), transmission (T), scattering, and extinction parameters are the key indicators for optics, whereas carrier generation, transport, recombination, short circuit current, and quantum efficiency drive the solar cell, as shown in Figure 16. Interactions of plasmonic materials with solar cells have been computationally modeled using the following numerical methods: (1) finite difference time-domain (FDTD) [247], (2) finite element analysis (FEA) [248,249], (3) discontinuous Galerkin time-domain (DGTD) [143], (4) volume integral formulation [250], (5) surface integral formulation [251], (6) rigorous coupled wave analysis [252].

**Figure 17:**
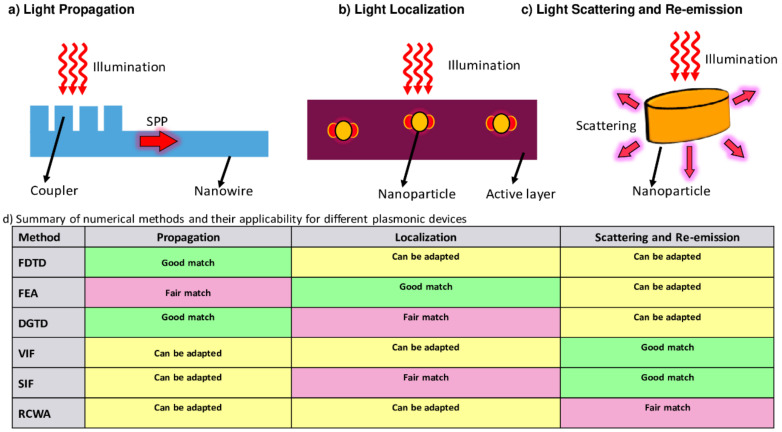
Light mechanisms involved in modeling plasmonic solar cells and comparison of modeling methods required.

Multiphysics problem can be simulated using commercial software packages above numerical techniques such as FEA-based COMSOL [253], FDTD-based LUMERICAL [254] and FDTD-based ATLAS [255]. Moreover, there are also isolated packages for plasmonics (such as MNPBEM [256], MEEP [257], and PyGDM [258]) and solar cells (such as SCAPS [259], AMPS [260], PC1D/PC2D [261], and QUOKKA [262]), which can be coupled together to perform optimization problems using multiproperty optimization methods [263]. Recently, the research community has also focused on machine learning approaches for developing inverse design of photonic systems [264,265,266]. Mathematically, there are three standard categories for addressing functional plasmonic and photonic structures. They depend on light localization, propagation, and scattering mechanism, as shown in Figure 17 [267]. Accordingly, each light mechanism requires a dedicated numerical method to capture the physics of the process, as compared in Figure 17d.

### 9.1. Device Designs Using Propagation of EM Waves

It includes systems with light confinement in 1 or 2-dimension space in subdiffraction limits. It is comprised of Surface Plasmon Polaritons (SPP) in plasmonic waveguides and nanoparticle arrays as shown in Figure 17a [268]. In these systems, the main structures are the waveguide modes using their resonance frequency, electric field intensity, and losses. The coupling of SPPs with device layers is also another area important for optimization [269,270]. FDTD and DGTD methods for solving Maxwell’s equations are best suited for simulating propagation-based plasmonic devices. They are capable of providing clear information for systems with light confinement in 1 or 2-dimensions space like decay kinetics in lossy multilayer channels and waveguides [271].

### 9.2. Plasmonic Systems Based on Light Localization

It confines the light in the three-dimensional region under the diffraction limit. Research is primarily focused on near-field enhancement i.e., towards light concentration using gratings on top of devices or nanoparticles in the active layer to enhance photonic absorption [272]. Figure 17b shows the schematic of a solar cell active layer equipped with nanoparticles and the associated electric field enhancement. Surface-enhanced Raman scattering and infrared absorption characterization techniques are used for measuring field confinement [273]. Nanophotonic devices relying on the localization effect include solar cells [274,275], gas sensors [276] and thermal imaging [277]. Finite Element Analysis is the preferred choice for localization problems due to its accurate prediction of resonance frequencies and near-field properties especially for irregular geometries in. DGTD and SIF provide fair results and moderately covers the key performance areas. Other methods such as VIF, FDTD, and RCWA show relatively low accuracy in assessing near-field intensity [272].

### 9.3. Problems Related to Light Scattering

It typically includes the interplay of nanostructure and electromagnetic field propagation. Nanoparticles suspended in the dielectric medium are an example of such systems. The key figure of merit for these nanoparticles are scattering, absorption and extinction coefficient [278]. For larger systems with nanostructure periodicity, the response variables become transmission, reflection, and absorption. Electron energy loss spectroscopy (EELS) utilizes the concept of inelastic scattering for assessing the atomic and electronic properties [279]. Light scattering problems are best solved by integral methods such as VIF, SIF, and RCWA. SIF methods are capable of dealing with bigger photonic systems than its counterpart VIF methods. RCWA, in contrast, shows efficient performance for periodic structure however is limited by simpler geometries [267].

## 10. Summary

The recent advancements in plasmonics for inorganic/organic PVs are summarized in this work. For the development of thin-film solar cells, advanced light-trapping techniques are key to obtain higher efficiency at lower costs. In this study, we summarize the relevant knowledge that can be extracted to unveil plasmonic processes and mechanisms. It is important to understand that electrical parameters should be studied in conjunction with photophysics to ensure that the improvement of plasmonic absorption leads to enhanced efficiency of charges and device performance. Nanostructures, when incorporated into PV devices, enhance antireflection, optical absorption, and wavelength spectrum and help in successful excitons dissociation. Moreover, it has been shown that the plasmonic effect is highly dependent on the distance between the nanostructures and the surface of the absorption layer. Furthermore, the positioning of nanostructures inside or between different layers in PV devices impacts device performance because of the different mechanisms induced. Similarly, it has been reported that broadband absorption can be obtained by different strategies such as hybrid approaches, i.e., combining different metallic or nonmetallic functional nanomaterials and nanostructures. For trapping light in the subwavelength region, nanostructures with specific sizes and geometries are anticipated to obtain the maximum possible PCE. There is much yet to explore in plasmonic nanoparticle applications in thin-film solar cells. Researchers can systematically work on the plasmonic behavior of metallic and hybrid nanoparticles of different sizes, morphologies, and densities in different layers of thin-film solar cells to obtain an optimized set of parameters for maximum light harvesting and subsequent conversion to power.

## Figures and Tables

**Table 1:** Summary of plasmonic nanoparticle applications in organic solar cells and effects on cell performance.

Ref.	Structure	Spatial Arrangement	J_sc_ (mA/cm^2^)	J_sc_ Enhancement (%)	Efficiency (ɳ %)	Efficiency Increase (%)	Mechanism
[13]	ITO/PEDOT:PSS/P3HT: PCBM/embedded Ag (NPs)/Al	Embedded in active layer	8.67 to 10.64	18.2	3.19 to 4.21	~23	LSPR and light scattering
[189]	ITO/PEDOT:PSS/BHJ active layer with Ag (nanocluster, 40 nm)/TiOx/Al	Ag nanoclusters embedded in active layer	10.79 to 11.61	7.6	6.3 to 7.1	11.3	Improved absorption by light scattering, increasing the optical length
[140]	ITO/PEDOT:PSS (30 nm)/ P3HT:PCBM (220 nm):Ag (sphere and prism)/Ca (80 nm)/Al (100 nm)	Ag NPs and nanoprisms embedded in active layer	10.61 to 8.99	18	3.6 to 4.3	16.3	Broadband resonance due to excitation of versatile plasmonic resonances
[190]	ITO/PEDOT:P:SS/BHJ active layer with Au NPs (70 nm) (truncated octahedraon)/TiOx/Al	Embedded in active layer	10.65 to 11.16	4.5	4.54 to 6.45	30	Light absorption caused by the light scattering of Au NPs in the active layer
[191]	ITO/PEDOT:PSS/PTB7:PC70BM/Au nanospheres (30 nm)/TiOx/Al	Au nanospheres embedded into active layer	15.31 to 15.70	2.5	7.02	6.7	Increased light absorption by light scattering of embedded Au nanospheres
[192]	ITO/rGO:ZnO/P3HT:PCBM:Ag/Au (NPs or NRs)/MoO3/Ag	Embedded Ag/Au NPs and NRs	10.99 to 12.21	10	3.77 to 4.85	~28	LSPR and light scattering
[193]	PCDTBT:PC71BM: WS2-Au	Embedded in active layer	10.6 to 12.3	14	5.6 to 6.3	~13	LSPR
[194]	ITO/PEDOT:PSS/PBDT-TS1:PC71BM/Au nanostars in active and PEDOT/Ca (ZnO)/Al	Embedded in active and HTL	18.37 to 19.24	5	9.97 to 10.50	~5	Plasmonic asymmetric modes of Au NSs transferred the optical power in ETL to active layer and improved the active layer absorption
[195]	PEDOT/Au nanorods (NRs) @ SiO2/PTB7:PC71BM	Sandwiched between CTL and active layer	16.5 to 21.2	22	7.52 to 9.55	~28	Scattering, LSPR
[10]	PCDTBT:PC71BM/Au NRs in TiOx	Incorporated Au NRs in back ETL	10.87 to 12.03	9.6	5.96 to 6.75	~13	Backward scattering
	PTB7:PC71BM/Au NRs in TiOx	Incorporated Au NRs in back ETL	16.27 to 17.17	5.2	7.43 to 8.01	~8	Backward scattering
[196]	PTB7:PC71BM/ZnO@carbon nanotubes (CNT)-Au (ETL)	Embedded ZnO@CNT-Au as ETL	16.18 to 16.81	4	7.0 to 7.9	~13	Forward scattering
[197]	PCDTBT:PC71BM/ZnO (ETL)	Embedded Au arrows in ETL	14.70 to 17.40	15.5	6.14 to 7.82	~27	Forward scattering, LSPR
[198]	PBDTTT-CF:PC71BM/ZnO (ETL)	Embedded Au NPs in ETL	14.49 to 15.81	8.3	6.67 to 7.86	~18	Forward scattering, LSPR
[199,200]	P3HT:ICBA/WO3 (HTL)	Embedded Cu NPs in rear HTL	8.71 to 11.79	26	4.65 to 6.38	~37	Backward scattering
[201]	P3HT:ICBA/WO3 (HTL)	Embedded Ag–Au bimetallic NPs in rear HTL	7.91 to 11.01	28	4.57 to 6.55	~43	Backward scattering
[87]	PTB7:PCBM/PEDOT/Ag nanodot array/ITO	Sandwiched between HTL and anode	17.43 to 23.26	25	7.70 to 10.72	~39	LSPR, forward scattering
[12]	Ag networks/ZnO/PCDTBT:PCBM/MoO3/Ag oblate NPs/anode	Embedded Ag oblate NP array between HTL and anode	9.32 to 11.37	18	5.22 to 6.01	~13	Hybridization of LSPR and plasmonics gap
[202]	PTB7:PC71BM/ZnO/Au NPs/ITO	Incorporated Au NPs between ETL and cathode	15.53 to 15.69	1	6.75 to 7.27	~7	MDM absorber
[203]	PTB7:PC71BM/nano–biohybrid/ZnO/ ITO	Incorporated Ag prisms-LHCII between the active layer and ETL	16.01 to 17.99	11	9.03 to 10.57	~17	LSPR
[204]	PBDTTT-C:PC60BM/Au NPs/PEDOT/ITO	Incorporated Au NPs ~15 nm between the active and HTL	10.62 to 11.74	10	4.78 to 5.52	~15	LSPR
[205]	ITO/ZnO/P3HT:PC61BM/MoO3/Al	Reference Ag NWs between cathode and ETL Ag NWs between ETL and active layer	8.13 to 9.87	17.6	3.10 to 4.05	~23	LSPR
[41]	ITO/ZAZ/P3HT:PC61BM/PEDOT:PSS/Ag	Applied ZnO/AgNWs/ZnO (ZAZ) as transparent electrode	9.75 to 11.6	16	3.16 to 3.53	~12	Higher transmission above 450 nm
[39]	P3HT:PC61BM/PEDOT/ Au (flat or grating)	Applied Au grating as rear electrode	6.13 to 6.83	10.2	3.03 to 3.53	~16	SPP, photonic waveguide mode
[206]	ITO/PEDOT:PSS:Ag Nanoparticles (NPs) (57 nm)/CuPc/C60	Embedded Ag NPs into photoconductor transport layer	4.01 to 5.01	20	0.663 to 0.925	~28	LSPR

**Table 2:** Summary of studies in which plasmonic nanoparticles were applied in metal-halide perovskite solar cells along with the resulting PV performance.

Ref.	Structure	J_sc_ (mA/cm^2^)	J_sc_ Enhancement (%)	Efficiency (ɳ %)	Efficiency Increase (%)	Mechanism
[207]	ITO/PEDOT:PSS/MAPbI_3_/Ag NPs (79 nm)/PCBM/LiF/Al	19.89 to 24.41	18.5	11.63 to 13.46	13.6%	Improved J_sc_ and overall device performance due to enhanced absorption via LSPR and light optical path length increase.
[208]	ITO/Au NPs (120 nm):QD-CsPbBr_3_/PEDOT:PSS/MAPbI_3_/C_60_/Ag	20.6 to 22.5	9	8.53 to 10.9	27.8	LSPR excitation and light scatterring.
[209]	ITO/PEDOT:PSS/MAPbI_3_/PCBM/Ag (nanocubes)/BCP/Ag	19.5 to 21.4	9	11.9 to 13.3	10.5	Plasmonic Ag nanocubes coupling with Ag back electrode.
[210]	ITO/TiO_2_/ZrN/SiO_2_ NPs (75 nm core/40 nm shell)/MASnI_3_/Spiro-OMeTAD/Au	27 to 40.3	33	12.9 to 20	35.5	Attributed to the enhancement in the plasmonic surface plasmon directivity by the dielectric shell.
	ITO (150 nm)/TiO_2_ (40 nm)/TiN NPs (100 nm)/MASnI_3_ (350 nm)/Spiro-OMeTAD (200 nm)/Au (100 nm)	27 to 36.91	27	12.9 to 18.2	29	Absorption enhancement due to NP plasmonic effect acting as wave guide to direct sunlight by LSPR, forming SPPs at the air–TiN interface.
	ITO (150 nm)/TiO_2_ (40 nm)/ZrN NPs (100 nm)/MASnI_3_ (350 nm)/Spiro-OMeTAD (200 nm)/Au (100 nm)	27 to 34.2	21	12.9 to 16.6	22.3	Plasmonic resonance enhancement at NIR wavelengths.
[211]	FTO/TiO_2_ (50 nm)/Al_2_O_3_ (130 nm) with Au(80 nm)@SiO_2_ (8 nm) + MAPbI_3_/Spiro-OMeTAD/Ag	14.76 to 16.91	13	10.7 to 11.4	6	Enhanced photocurrent due to enhanced light absorption and plasmonic localized heating.
[212]	FTO/Ag@TiO_2_/Al_2_O_3_ + MAPbI_3_/Spiro-OMeTAD/Ag	17.3 to 20.2	14.35	11.4 to 13.5	16	Photocurrent improvement due to highly polarizable metallic NPs.
[213]	FTO/c-TiO_2_/m-TiO_2_/Au-Ag alloy NPs (popcorn-shaped)/MAPbI_3_/Spiro-OMeTAD/Ag	15.51 to 16.46	6	8.9 to 10.3	15.7%	Plasmonic popcorn NPs led to faster charge transfer at TiO_2_–perovskite interface, resulting in increased PCE.
[214]	FTO/TiO_2_/SnO_2_/CsFAMAPbI_3_Br_3_/Ag NR (buffer layer)Spiro-OMeTAD/Au	21.08 to 22.18	5	18.50 to 20.29	9	Ag NRs increased the absorption by the LSPR effect.
[215]	ITO/TiO_2_/Au@TiO_2_ (NR)/MAPbI_3_/Spiro-OMeTAD/Au	20.78 to 22.27	7	15.76 to 16.35	20.10	Facilitated carrier transfer or separation in the presence of plasmonic NPs.
[216]	FTO/PEDOT:PSS + Ag NPs/MAPbI_3_/PCBM/Al	15.06 to 15.47	3	4.17 to 5.58	25.3	Plasmons induced enhanced absorption and superior photogenerated carrier separation and transport via the Ag NPs in the perovskite active material.
[217]	FTO/c-TiO_2_/TiO_2_ (nanocolumns, NC)/Cs_0_._05_(FA_0_._83_MA_0_._17_)_0_._95_Pb(I_0_._83_Br_0_._17_)_3_/SpiroOMeTAD/Au	19.27 to 20.19	4.6	15.31 to 16.38	6.5	TiO_2_ NCs improved the performance of perovskite halide solar cells in terms of charge transport, light harvesting, and stability.
[218]	FTO/c-TiO_2_/Au@TiO_2_ NPs embedded in p-TiO_2_/MAPbI_3_/Spiro-OMeTAD/Ag	17.40 to 23.12	25	12.59 to 18.24	44	Improvement due to exciton generation rate, enhanced exciton dissociation probability, and efficient carrier transfer/collection induced by the LSPR effect.
[219]	ITO/ZnO/MAPbI_3_/Au (nanostars)/Spiro-OMeTAD/Ag	17.43 to 18.21	4.3	11.98 to 13.97	14	Absorption improved by Au NSs because of SPR and backscattering effects.
[220]	FTO/ZnO/ZnO NR/MAPbI3/spiro-OMeTAD/Au	18.07 to 20.56	12.1	14.51 to 16.77	~14	LSPR.
[208]	120AuNPs:quantum dots (QD)-CsPbBr_3_/PEDOT:PSS/MAPbI_3_	20.6 to 22.5	8.4	8.53 to 10.9	~27.8	LSPR excitation by resonance interaction.
[221]	ITO/PEDOT:PSS/CH_3_NH_3_PbI_3_/PC_61_BM/Al	16.70 18.15 to	8	10.54 to 11.74	~10.22	Subwavelength antenna due to LSPR excitation.

**Table 3:** Summary of research in which plasmonic nanoparticles were applied in silicon solar cells.

Ref.	Silicon Solar Cell	Plasmonic Type	Position	Achievements
[222]	Amorphous silicon (a-Si) thin-film solar cell	SiO_2_ nanoparticles (70 nm)	Front	Increase in current short-circuit density of 21%; increase in conversion efficiency of 18%.
		Ag hemispheres (110 nm)	Rear	
[223]	a-Si solar cell	Double sided plasmonic bimetallic (Al–Cu) nanograting Al (60 nm width) Cu (50 nm width)	Front	Improvement in absorption of 40% and in J_sc_ of 22.30 mA/cm^2^ (compared with 16.46 mA/cm^2^ without grating).
[162]	Crystalline Si solar cell with TiO_2_ as ARC	Ag NPs (90 nm)	Front	Conversion efficiency increased from 9.53% to 16.04%, which was attributed to plasmonic effect.
[224]	Planar silicon solar cells with Al_2_O_3_ layers	Random-sized Ag NPs (20–140 nm)	Front	EQE increased by 19.2% at 700 nm, and PCE by 20%, compared with the reference Si solar cell without NPs.
[225]	Textured silicon solar cells with up-conversion and plasmonic scattering	Indium NPs (7 nm) in SiO_2_ layer	Front	Conversion efficiency increased from 14.45% (reference cell) to 15.43%.
[226]	Thin-film a-Si	Ring-shaped Ti nanoparticles	Front	Absorption improved by 40% from 300 to 700 nm compared with the reference.
[227]	Aluminum back surface field (BSF) Si solar cell	ITO nanoparticles scattered in SiO_2_ layer (10–90 nm)	Front	Efficiency improved by 33.27%.
[228]	Silicon heterojunction solar cells	Au nanoparticles (90 nm)	Front	Increase in short-circuit current of 15%.
[229]	a-Si/c-Si heterojunction solar cells	ITO nanoparticles (75 nm)	Rear	Increase in current from 32.8 mA/cm^2^ to 35.1 mA/cm^2^. Increase in efficiency from 13.74% to 15.22%.
[230]	Silicon heterojunction solar cells	Ag nanowire contacts (4 µm pitch)	Front	Increase in power conversion efficiency from 15.0% to 16.0%.
[231]	Textured silicon solar cell	Ag–Al nanoparticles in SiON matrix (average~115 nm)	Front	Increase in photocurrent from 26.27 mA/cm^2^ to 34.61 mA/cm^2^
[232]	Crystalline silicon solar cells	TiN nanoparticles (100 nm)	Front	Increase in absorption of 20%.
[233]	Aluminum BSF Si solar cell	Al_2_O_3_/In NPs (17.7 nm)/TiO_2_ antireflective coating	Front	Conversion efficiency increased from 10.96% to 16.93%.
[234]	Microcrystalline-Si solar cells	Plasmonic nanoshells of silica and gold (shell thickness 30 nm and core radius 50 nm)	Embedded in Si active layer	Increase in photocurrent of about 21%.
[235]	ZnO/p-silicon heterojunction cell	Silver nanoparticles (<10 nm)	Front	J_sc_ increased from 2.05 to 11.67 mA/cm^2^.
[236]	Thin Si solar cells	In NPs (17.7 nm)	Front	Short-circuit current improved by 31.88% and conversion efficiency improved by 32.72%.
		Ag NPs (100 nm)	Rear	
[237]	a-Si p–i–n solar cells	Au NPs (200 nm)	Front	Current density increased from 9.34 to 10.1 mA/cm^2^, and efficiency increased from 4.28% to 5.01%.
[238]	Microcrystalline silicon solar cell	Ag NPs (100 nm)	Front	Efficiency improved by 2.8%.
[239]	Passivated emitter rear totally-diffused (PERT)	Ag NPs (28 nm) + Si0_2_ and rear metal reflector	Rear	EQE improved by 400%, and J_sc_ by 16%.
[240]	Bifacial crystalline Si solar cells	Ag NPs (220 nm)	Front and Rear	EQE improved by 700%.
[145]	Passivated emitter rear locally-diffused (PERL)	Ag NPs (12 nm)	Front	EQE improved by 700%, and J_sc_ by 19%.
[241]	Planar crystalline silicon solar cells	Ag NPs (62 nm)	Front	Increase in efficiency by 35.2%, from 11.2% to 15.2%.
[242]	Si-Schottky barrier solar cells	Ag NP (19.7 nm)	Front	J_sc_ increased from 13.7 to 19.74 mA/cm^2^ (i.e., by 43.7%).
[243]	Si-based metal–insulator–semiconductor (MIS) Schottky junction solar cells	Nanoporous Si	Front	J_sc_ increased from 0.43 to 5.52 mA/cm^2^ (i.e., by 92.2%) due to reflection reduction and the passivation provided by nanoporous Si.
[243]	Si-based metal–insulator–semiconductor (MIS) Schottky junction solar cells	Nanoporous Si + Ag NPs	Front	J_sc_ increased from 0.43 to 8.07 mA/cm^2^ (i.e., by 94.6%) due to the small size of the AgNPs, SPR effects, and the improved electrical conduction of the nanoPS layers.
[185]	p–i–n a-Si:H solar Cell	SiO_2_ nanocone	Front	J_sc_ increased from 11.4 to 17.5 mA/cm^2^ (i.e., by 34.5%) due to suppression of reflection by nanodomes, which was due to the formation of a graded refractive index profile.
[244]	n–i–p a-Si:H Solar Cell	Ag back contact with patterned holes (225 nm)	Rear	J_sc_ increased from 9.86 to 12.5 mA/cm^2^ (i.e., by 26%) due to the periodic nanostructures on the back contact of an n–i–p a-Si:H solar cell (i.e., enhancing the red-response of the device).
[245]	Heterojunction silicon solar cell	SiO_x_ (70 nm) as ARC	Front	J_sc_ increased from 34.1 to 40.5 mA/cm^2^ (i.e., by 16%) due to the double-layer AR coating instead of single-layer.

## Data Availability

Not Applicable.

## Abbreviations

µc-Si:H	Microcrystalline silicon
1D	One-dimensional
2D	Two-dimensional
3D	Three-dimensional
Al-BSF	Aluminum back surface field
AAM	Anodic alumina membrane
ARC	Antireflection coating
AFM	Atomic force microscopy
a-Si:H	Hydrogenated amorphous silicon
AZO	Alumina-doped zinc oxide
CdS	Cadmium sulfide
CdTe	Cadmium telluride
CIGS	Copper indium gallium diselenide
c-Si	Crystalline silicon
CTL	Charge carrier transport layer
DNA	Deoxyribonucleic acid
DPN	Dip-pen lithography
EBL	Electron beam lithography
EM	Electromagnetic
FIB	Focused ion beam
LIL	Laser interference lithography
LSPP	Localized surface plasmon polariton
LSPR	Localized surface plasmon resonance
LSP	Localized surface plasmon
MCPS	Microcone patterned substrate
NIL	Nanoimprint lithography
NPs	Nanoparticles
NSL	Nanosphere lithography
OPV	Organic photovoltaics
PCE	Power conversion efficiency
PV	Photovoltaics
QCS	Quasicrystal structure
RCWA	Rigorous coupled wave analysis
SCS	Scattering cross-section
SECM	Scanning electrochemical microscopy
SEM	Scanning electron microscopy
SERS	Surface enhanced Raman spectroscopy
SPP	Surface plasmon polariton
SPR	Surface plasmon resonance
SSD	Solid-state dewetting
SThM	Scanning thermal microscopy
TCO	Transparent conductive oxide
TEM	Transmission electron microscopy
TiN	Titanium nitride
TiO_2_	Titanium oxide
TW	Terawatt
UV	Ultraviolet
ZrN	Zirconium nitride
λ	Wavelength
Λ	Periodic structure
